# A description of the hepatitis B virus genomic background in a high-prevalence area in China

**DOI:** 10.1186/1743-422X-11-101

**Published:** 2014-05-31

**Authors:** Xiaoming Chen, Jie Gao, Zhaohua Ji, Weilu Zhang, Lei Zhang, Rui Xu, Jingxia Zhang, Fei Li, Shi Li, Shijie Hu, Lei Shang, ZhongJun Shao, Yongping Yan

**Affiliations:** 1 Department of Epidemiology, School of Public Health, Fourth Military Medical University, No. 17, Changle west RD, Xi’an 710032, China; 2 Department of Cardiology, Xijing Hospital, The Fourth Military Medical University, No. 17, Changle west RD, Xi’an 710032, China; 3 Department of Otolaryngology, Xijing Hospital, The Fourth Military Medical University, No. 17, Changle west RD, Xi’an 710032, China; 4 Institute Of Neurosurgery, Xijing Hospital, The Fourth Military Medical University, No. 17, Changle west RD, Xi’an 710032, China; 5 Department of Statistics, School of Public Health, Fourth Military Medical University, No. 17, Changle west RD, Xi’an 710032, China

**Keywords:** Hepatitis B virus, Consensus sequences, Clinically relevant mutations

## Abstract

**Background:**

Hepatitis B (HB) is an important disease worldwide. Almost 350 million people are positive for Hepatitis B virus surface antigen (HBsAg), and one-third of them live in China. According to a nation-wide serosurvey in China in 2006, the prevalence of HBsAg was higher in Northwest China than in other areas. However, the epidemic HBV strains in this area are poorly studied.

**Results:**

In this study, 242 complete hepatitis B virus (HBV) genome sequences were obtained from HBV asymptomatic carriers in major cities of Northwest China. The 242 HBV sequences clustered into genotypes B, C and D. Through comparison of the genotype consensus sequences, 158 genotype-dependent positions were observed in P, S and X ORFs. Clinically relevant mutation screening in this study revealed that no HBV antiviral drug resistance mutations were observed and the vaccination failure mutations were heavily underrepresented.

**Conclusions:**

The role of genotype D strains in HBV prevalence should not be ignored in Northwest China. Due to low prevalence of vaccination failure mutations, it can be inferred that the genotype B, C and D strains in Northwest China may have less likelihood of vaccine escape.

## Background

Hepatitis B virus (HBV) infection is an important global disease. It is estimated that almost 350 million people are chronically infected, and the virus causes approximately 1 million deaths annually [1,2]. A nation-wide serosurvey in 2006 in China showed that in the population aged 1–59 years old, the prevalence of HBsAg was 7.2% overall and varied in different areas [3]. In Northwestern China, due to poor HBV knowledge, delayed vaccine inoculation or other unknown reasons, the HBV prevalence is over 8.3% according to our study (unpublished), which is much higher than in other areas. However, the epidemic HBV strains in this area of China are poorly studied. For example, among the 1535 complete Chinese HBV genomes published in the NCBI database, only 18 (1.17%) were isolated in Northwest China. Therefore, to gain insights into HBV prevalence in this highly epidemic area, 242 complete HBV genome sequences isolated from this region represented a genomic background for hepatitis B virus, and this genomic background may provide an important reference for studies of HBV evolution or for further clinical studies. To our knowledge, this is the first comprehensive description of the HBV genomic background in this highly epidemic area of China.

## Methods

### Study subjects

From September 2006 to August 2012, 285,858 residents undergoing a physical examination at the physical examination department of the local centers for disease prevention and control were enrolled into this study from major cities in Northwest China, i.e., Lanzhou City of Gansu Province, Xi’an City of Shaanxi Province, Xining City of Qinghai Province, Urumuqi City of the Xinjiang Autonomous Region and Yinchuan City of the Ningxia Autonomous Region. HBV serologic markers were tested using commercially available enzyme-linked immunosorbent assay kits (Abbott Laboratories, USA; Beijing Wantai Co., Ltd, Beijing). Information about HB-related medical history was collected by trained investigators. The serum viral load was measured with qualitative assays (Roche Ltd., Switzerland). Only patients positive for HBsAg, having no HB-related medical history or typical clinical symptoms, and with HBV DNA ≥ 10^5^ IU/ml were enrolled. Approval was obtained from the local and Fourth Military Medical University institutional ethics committee before the study, and informed consent was obtained from each individual. All serum samples were collected and stored at −80°C before use.

### Complete HBV genome sequencing

HBV DNA was extracted from 200 μL serum samples using DNA/RNA extraction kits (Beijing Tiangen Co., Ltd, Beijing). HBV DNA genomes were obtained by separately amplifying three HBV segments: segment I (nt 56–756), primers 5’-CTGCTGGTGGCTCCAGTT-3’ (sense primer, nt 56–73) and 5’-CAATACCACATCATCCAT-3’ (anti-sense primer, nt 756–739); segment II (nt 736–1889), primers 5’-TATATGGATGATGTGGTATTGGG-3’ (sense primer, nt 736–758) and 5’-ACCCAAGGCACAGCTTGG-3’ (anti-sense primer, nt 1889–1872); and segment III (nt 1806–195), primers 5’-CCAGCACCATGCACTTTTT-3’ (sense primer, nt 1806–1824) and 5’-CGAGCAGGGGTCCTAGGA-3’ (anti-sense primer, nt 195–177). PCR procedures were set as previously described [4]. For each sample, three groups of PCR products were cloned into the pCR4-TOPO vector (Invitrogen, Shanghai), which was used to transform Top10 electro-competent *Escherichia coli* cells (Invitrogen, Shanghai) by electroporation. Colonies were screened using M13 universal primers. M13 PCR products of the correct size were sequenced [5]. Furthermore, for each sample, the three acquired sequences were analyzed and integrated with Codoncode Aligner Software (version 4.0.3) into a complete HBV genome.

### Molecular evolutionary analyses

All 242 HBV sequences generated in this study were manually edited by visual inspection and multiply aligned with reference HBV sequences (GenBank accession number: Genotype A: AY233274; Genotype B: AB246341; Genotype C: AB111946; Genotype D: EU594396; Genotype E: AB032431; Genotype F: EU670262; Genotype G: AF160501) using Bioedit software (version 7.0). A phylogenetic tree was constructed using the UPGMA method. The reliability of the pairwise comparison and phylogenetic tree analysis was assessed by bootstrap resampling with 1000 replicates. Phylogenetic and molecular evolutionary analyses were conducted using MEGA (version 5.05) [6]. Consensus sequences were constructed using the Mutation Master Server (http://cagt.bu.edu/page/MutationMaster_about) [7]. Consensus sequences of different genotypes were compared to determine genotype-dependent nucleotide positions among different genotypes. Clinically relevant mutations were also analyzed, as guided by previous clinical studies.

### Statistical comparisons

Significant differences among different genotypes were calculated using the χ2 test, Fisher’s exact probability test and Student’s t-test where applicable. Two-tailed P values ≥ 0.05 were considered statistically significant. The R Project software version 2.14.2 was utilized for statistical calculations.

### GenBank accession number

GenBank accession numbers of the acquired sequences were KC774180-KC774242, KC774280-KC774377, KC774393-KC774434 and KC774461-KC774499.

## Results

### Demographic information

**Figure 1:**
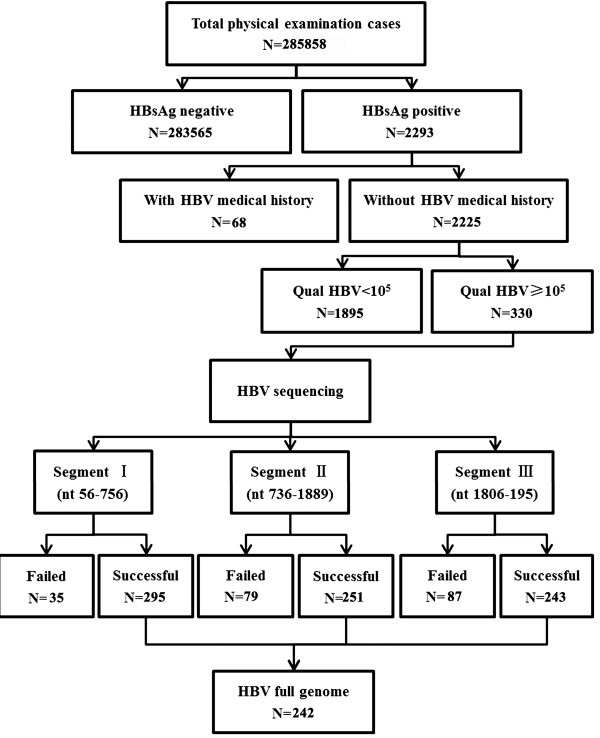
The processes of case enrollment and complete HBV genome amplification.

Among 2293 HBsAg-positive patients without HB-related medical history, 330 were identified with HBV ≥10^5^ IU/ml, and all patients were positive for Hepatitis B Virus E antigen (HBeAg). Serum samples from these 330 patients were subjected to amplification of the full-length HBV genome, and 242 full-length HBV genomes were finally obtained (Figure 1). Sixty-seven full-length HBV genomes were obtained from the patients of Lanzhou, 27 from Urumuqi, 78 from Xi’an, 56 from Xining, and 14 from Yinchuan (Table 1). There was no significant difference in the age, M/F ratio or clinical data with respect to case locations.

**Table 1:** Demographic and clinical information for the patients in this study

**City**	**No. of cases**	**Age, yr (mean ± sd)**	**M/F ratio**	**ALT (U/L)**	**AST (U/L)**
**Lanzhou**	**67**	**29.63 ± 13.78**	**42/25**	**42.65 ± 36.37**	**34.27 ± 25.70**
**Urumuqi**	**27**	**28.74 ± 13.75**	**10/17**	**48.79 ± 56.16**	**43.21 ± 59.04**
**Xi’an**	**78**	**28.52 ± 12.88**	**40/38**	**43.81 ± 41.12**	**39.33 ± 40.91**
**Xining**	**56**	**30.05 ± 14.59**	**29/27**	**42.77 ± 33.34**	**38.63 ± 21.74**
**Yinchuan**	**14**	**32.29 ± 15.79**	**8/6**	**29.00 ± 19.29**	**24.15 ± 12.22**
**Total**	**242**	**28.74 ± 13.14**	**132/110**	**43.89 ± 46.93**	**39.36 ± 45.42**

### HBV genotypes and serotypes

**Figure 2:**
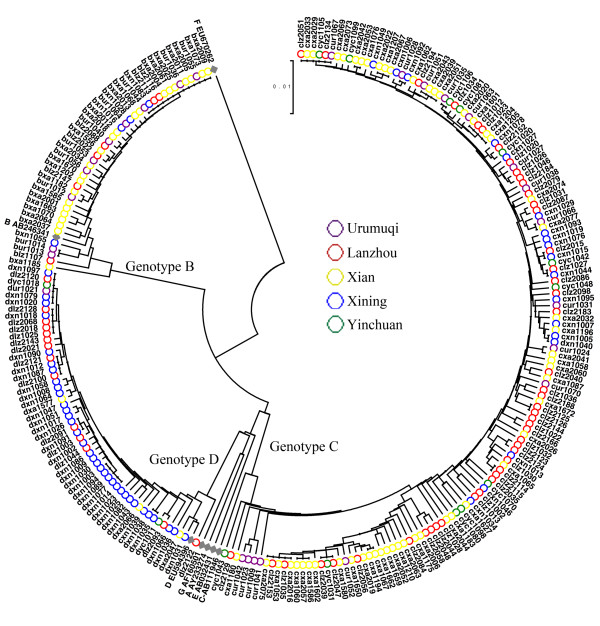
**Phylogenetic tree of the complete HBV genomes in this study generated by the UPGMA method.** The reliability of pairwise comparison and phylogenetic tree analysis was assessed by bootstrap resampling with 1000 replicates. The reference sequences of different genotypes were retrieved from GenBank. The accession number was as follows: Genotype A: AY233274; Genotype B: AB246341; Genotype C: AB111946; Genotype D: EU594396; Genotype E: AB032431; Genotype F: EU670262; Genotype G: AF160501.

Phylogenetic analysis with GenBank reference sequences indicated three distinct clusters, corresponding to the HBV genotypes B, C and D (Figure 2). Among the 242 sequences generated in this study, genotype C (59.92%, 145/242) was the most frequently observed, followed by genotype D (22.31%, 54/242) and genotype B (17.77%, 43/242). The relationship between case locations and genotypes is displayed in Table 2.

**Table 2:** Relationship between complete HBV genome locations and genotypes

**City**	**No. of sequences**	**Genotype B**	**Genotype C**	**Genotype D**
**Xi’an**	**78**	**23**	**52**	**3**
**Lanzhou**	**67**	**6**	**47**	**14**
**Yinchuan**	**14**	**0**	**12**	**2**
**Urumuqi**	**27**	**10**	**16**	**1**
**Xining**	**56**	**4**	**18**	**34**
**Total**	**242**	**43**	**145**	**54**

In serotype analysis, the amino acids in the viral surface gene were checked in the manner of previous studies to determine the serotype of each strain. As a result, subtype adrq- (122 K + 127P + 134 F + 159 V + 160R + 177A +178P), adrq + (122 K + 127P + 134 F + 159A + 160R + 177 V +178P), adw2 (122 K + 127P + 134 F + 159A + 160 K + 177 V +178P), adw4q- (122 K + 127 L + 134 F + 159A + 160 K + 177 V +178P), ayr (122R + 127P + 134 F + 159A + 160R + 177 V +178P), ayw1 (122R + 127P + 134 F + 159A + 160 K + 177 V +178P), ayw2 (122R + 127P + 134Y + 159G + 160 K + 177 V +178P) and ayw3 (122R + 127 T + 134 F + 159G + 160 K + 177 V +178P) were observed in these sequences. Subtype adrq + (56.61%, 137/242) was the most frequently observed, followed by subtype ayw2 (23.97%, 58/242) and adw2 (16.12%, 39/242). In addition, there was no significant difference in the distribution of HBV serotypes with respect to genotypes (Table 3) [8].

**Table 3:** Relationship between complete HBV genome genotypes and serotypes

**Genotype**	**adr**		**adw**		**ayr**	**ayw**			**Total**
	**adrq-**	**adrq+**	**adw2**	**adw4**	**ayr**	**ayw1**	**ayw2**	**ayw3**	
**B**	**0**	**28**	**3**	**1**	**0**	**1**	**10**	**0**	**43**
**C**	**0**	**84**	**25**	**0**	**1**	**1**	**33**	**1**	**145**
**D**	**1**	**25**	**11**	**1**	**1**	**0**	**15**	**0**	**54**
**Total**	**1**	**137**	**39**	**2**	**2**	**2**	**58**	**1**	**242**

### Genotype consensus sequences

**Figure 3:**
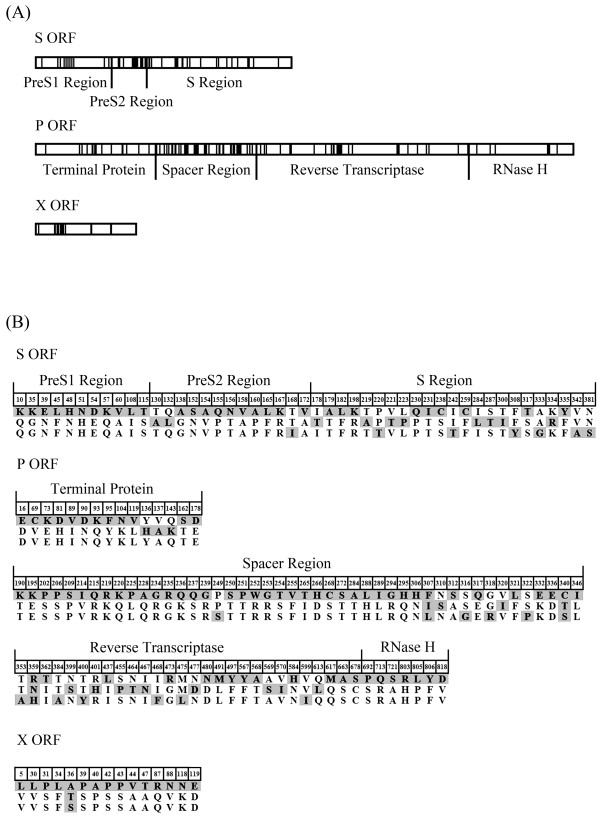
**Mapping of genotype-dependent positions. (A)** Locations of genotype-dependent positions in S, P and X ORFs. **(B)** Amino acid changes in the genotype-dependent positions. The genotype consensus sequences for genotype-dependent position comparisons were determined using the Mutation Master Server online, using an input of the complete HBV genotype B-D sequences as generated in this study.

Through comparison of the three genotype consensus sequences, 158 amino acid positions were found to be significantly different among the consensus sequences. Among these 158 identified genotype-dependent positions, 96 (60.76%) were located in the P ORF, 47 (29.75%) in the S ORF and 15 (9.49%) in the X ORF. No positions were found in the C ORF (Figure 3A).In the P ORF, 46 out of 96 (47.92%) genotype-dependent positions were located in the spacer region, followed by 28 (29.17%) in reverse transcriptase, 15 (15.63%) in terminal proteins and 7 (7.29%) in RNase H. In the S ORF, 23 out of 47 (48.93%) genotype-dependent positions were located in the S region, followed by 11 (23.40%) in the preS1 region and 13 (28.26%) in the preS2 region. In the X ORF, 11 out of 15 (73.33%) genotype-dependent positions were located in the N-terminal region (first 50 amino acids), while only 4 (36.36%) were located in the C-terminal region. Interestingly, in 112 of the 158 (70.89%) positions, the genotype B consensus sequence showed differences from the other consensus sequences of genotypes C and D, while the latter two were identical to each other. This finding agreed with the higher divergence between genotypes B and C/D (Figure 3B).

### Clinically relevant mutations

To investigate the biological and clinical characteristics of HBV sequences, all the sequences in this study were screened for clinically relevant mutations that were reported in previous studies. In the P ORF, all sequences were screened for mutations that were reported in previous studies to be associated with drug resistance: rtL80V/I, rtL180M and rtM204V/I mutations, associated with LMV resistance; rtA181T and rtN236T mutations, associated with ADV resistance; rtS184G, rtS202I and rtM250V mutations, associated with ETV resistance; the rtA194T mutation, associated with TDF resistance; and the rtM204I mutation, associated with LdT resistance [9,10]. However, none of these mutations were observed in the present study.

In the S ORF, preS1 deletion mutations were detected in 16 sequences, and preS2 deletion mutations were detected in 5 sequences. In addition, G129H/R mutations, which have been suggested to be associated with low antibody adherence, were found in the S region in eight sequences [11]. G145R mutations, which were reported to be responsible for vaccination failure, were found in five sequences. Furthermore, in the α determinant, which is vital to HBV antigenicity, mutations were also found in aa126, aa127, aa130, aa131, aa134 and aa143 (Table 4).

**Table 4:** List of clinically relevant mutations detected in the S ORF of the HBV complete genome sequences acquired in this study

	**Genotype B**	**Genotype C**	**Genotype D**	**Total number**
**No of sequences**	**43**	**145**	**54**	**242**
preS1 deletion mutation**	**0**	**5**	**11**	**16**
**preS2 deletion mutation**	**1**	**4**	**0**	**5**
**G129H/R**	**1**	**5**	**2**	**8**
**G145R**	**1**	**4**	**0**	**5**
**T126A/S**	**1**	**1**	**1**	**3**
**P127T**	**0**	**4**	**2**	**6**
**G130R**	**1**	**2**	**0**	**3**
**T131P**	**2**	**5**	**2**	**9**
**M134Y**	**11**	**31**	**14**	**56**
**S143T/L**	**8**	**20**	**11**	**39**

Mutations whose distributions varied significantly in different genotypes are indicated with “**” (P < 0.01).

The distribution of clinically relevant mutations in different genotypes was compared and is shown in Table 4. PreS1 deletion mutation in the S ORF was detected with a significantly different distribution among different genotypes. Genotype D sequences were shown to have significantly higher frequencies of preS1 deletion mutation.

## Discussion

HBV infection is an epidemic in China. In Northwest China, the prevalence of HBV infection is much higher compared to other areas. In previous HBV studies in Northwest China, phylogenetic analysis of the HBV S region was employed to reveal the HBV genotypes [12]. However, the complete characteristics of epidemic HBV strains have not been well studied, especially in those strains in asymptomatic carriers. In this study, 242 complete HBV genome sequences were isolated from asymptomatic carriers who underwent physical examinations in the local centers for disease prevention and control. Based on these sequences, consensus sequences of genotypes B, C and D were determined and compared with each other, and moreover, all the sequences were screened for clinically relevant mutations. This study, which presents the HBV genomic background of early stage infection, will contribute to the establishment of a reliable virus evolution history and provide vital genomic baseline references for further clinical studies.

In China, HBV genotypes B and C are suggested to be the major genotypes in the population [13]. Since the time Genotype D was first reported by Fan J *et al.* in the Qinghai-Tibet Plateau in 1997, it has never been considered a major genotype in China [5,14]. However, in this study, 54 of 242 (22.31%) HBV sequences were identified as genotype D in Northwest China, which was higher than expected. It can be inferred that genotype D may play a more important role in HBV prevalence in western China than previously expected.

The HBV genotype consensus sequence is important in establishing an HBV genotype sequence motif and inferring the genotype-dependent function of various HBV domains. Mutation Master is a reliable server that rapidly provides a visual display of consensus sequences and genetic variability, using multiple sequence alignments [7]. In this study, Mutation Master Server was used to analyze HBV sequences of different genotypes for consensus sequences and genetic variability. By comparing different genotype consensus sequences, we found that most of the genotype-dependent variability was concentrated in the spacer region, which has not previously been reported.

The P ORF and S ORF are the most important for the prevention and therapy of HBV infection. In the P ORF, 28 genotype-dependent positions were detected in the reverse transcriptase region, where there is a functional domain in which reverse transcription and synthesis of the second DNA strand occurs. Positions within this region may be influenced and selected during antiviral treatment by using nucleoside/nucleotide analogs, such as lamivudine [10]. The presence of HBV genotype-dependent variability in this therapeutic target region suggests the role of clinical treatment selection in the evolution of different HBV genotypes. In clinically relevant mutation screening analysis, no HBV antiviral drug resistance mutations were observed, which was consistent with a similar HBV genetic diversity study conducted in American blood donors [15]. Drug-resistant HBV variants may be inefficient in transmission and/or establishment of a chronic infection or may be underrepresented in the pool of HBV that is actively transmitted by sexual or parenteral routes [16].

In the S region, 23 genotype-dependent positions were detected, and 9 of these positions were located in the major hydrophilic region. The small S protein is encoded by the S region and is a major component of the viral envelope that plays an important role in the host immune response. The major hydrophilic region encompasses aa101-aa160 of the S protein and is exposed on the surface to both virions and subviral particles. This region is highly immunogenic and is potentially under the selective pressure of the immune system [17]. The presence of HBV genotype variability in this immune-dominant region suggests the role of immune selection in the evolution of different HBV genotypes. Deletion mutations in preS regions were much less prevalent (6.61%) compared to previously reported results in Chinese chronic HBV infections and HCC (20%) [18,19]. This observation is consistent with these mutations leading to impaired virus particle secretion and thus being negatively selected during the transmission of HBV. According to a similar study of HBV genetic diversity in American blood donors, the prevalence of the well-known neutralization escape mutation G145R in the HBV envelope protein was as high as 22% [15]. However, in contrast to those results, in our study, the G145R mutation was heavily underrepresented (2.07%), from which it can be inferred that the genotype B, C and D strains in Northwest China may have less likelihood of vaccine escape [20]. Furthermore, compared to the G145R mutation, several mutations within the major hydrophilic region were detected at high prevalence, such as M134Y and S143T/L. It can be inferred that these mutations may have been strongly selected in long-term infected carriers, in whom a strong antibody response develops [21].

This study has several limitations. First, the analysis was restricted to HBsAg-positive infections as detected by current blood HBV-screening assays. Consequently, infections by highly divergent variants that would not be detected by these assays would not be identified. Second, cases with low HBV qualification were not selected for complete HBV genome amplification by PCR due to amplification and sequencing accuracy, and moreover, a moderate proportion of cases were not able to be characterized due to failure of complete genome amplification by PCR. Third, bulk PCR product sequencing was performed in this study and therefore may not have detected cases of dual infection, minor populations of drug resistance or immune escape variants represented in viral quasi-species.

## Conclusions

In summary, the role of genotype D strains in HBV prevalence should not be ignored in Northwest China. Based on B, C and D genotype consensus sequences, genotype-dependent variability was frequently observed, and this variability might modulate hepatitis B-related clinical treatments and host antibody development. Among all detected clinically relevant mutations, the prevalence of confirmed vaccine-escaping mutations was low, and no HBV antiviral drug resistance mutations were observed.

## Competing interests

The authors declare that they have no competing interests.

## Authors’ contributions

LS, FL, YYP and SJH collected the blood samples and administered the questionnaires to the subjects in various CDCs. XMC, JG and ZHJ carried out the molecular genetic studies, participated in the sequence alignment process and drafted the manuscript. LZ carried out the HBV-related serum marker test. RX, WLZ and JXZ participated in the HBV complete genome amplifications. XMC carried out the integration and alignment of HBV sequences. All authors read and approved the final manuscript.
